# Metabolic engineering strategies for naringenin production enhancement in *Streptomyces albidoflavus* J1074

**DOI:** 10.1186/s12934-023-02172-5

**Published:** 2023-08-29

**Authors:** Suhui Ye, Patricia Magadán-Corpas, Álvaro Pérez-Valero, Claudio J. Villar, Felipe Lombó

**Affiliations:** 1 Research Group BIONUC (Biotechnology of Nutraceuticals and Bioactive Compounds), Departamento de Biología Funcional, Área de Microbiología, Universidad de Oviedo, Oviedo, Principality of Asturias, Spain; 2 Principality of Asturias, IUOPA (Instituto Universitario de Oncología del Principado de Asturias), Principality of Asturias, Spain; 3 ISPA (Instituto de Investigación Sanitaria del Principado de Asturias), Principality of Asturias, Spain

**Keywords:** Flavonoid, Polyphenol, Genome editing, Fermentation optimization, Malonate

## Abstract

**Background:**

Naringenin is an industrially relevant compound due to its multiple pharmaceutical properties as well as its central role in flavonoid biosynthesis.

**Results:**

On our way to develop *Streptomyces albidoflavus* J1074 as a microbial cell factory for naringenin production, we have significantly increased the yields of this flavanone by combining various metabolic engineering strategies, fermentation strategies and genome editing approaches in a stepwise manner. Specifically, we have screened different cultivation media to identify the optimal production conditions and have investigated how the additive feeding of naringenin precursors influences the production. Furthermore, we have employed genome editing strategies to remove biosynthetic gene clusters (BGCs) associated with pathways that might compete with naringenin biosynthesis for malonyl-CoA precursors. Moreover, we have expressed MatBC, coding for a malonate transporter and an enzyme responsible for the conversion of malonate into malonyl-CoA, respectively, and have duplicated the naringenin BGC, further contributing to the production improvement. By combining all of these strategies, we were able to achieve a remarkable 375-fold increase (from 0.06 mg/L to 22.47 mg/L) in naringenin titers.

**Conclusion:**

This work demonstrates the influence that fermentation conditions have over the final yield of a bioactive compound of interest and highlights various bottlenecks that affect production. Once such bottlenecks are identified, different strategies can be applied to overcome them, although the efficiencies of such strategies may vary and are difficult to predict.

**Supplementary Information:**

The online version contains supplementary material available at 10.1186/s12934-023-02172-5.

## Introduction

Naringenin, a citrus flavanone that belongs to the flavonoids polyphenols subgroup, is an important phytochemical displaying numerous pharmaceutical properties, such as antitumoral [1], neuroprotective [2], antidiabetic [3], hepatoprotective [4], anti-inflammatory [5] and antioxidant [6]. Besides, naringenin occupies a central position in the flavonoid biosynthesis pathway, as is used as the scaffold molecule for further enzymatic modifications involving diverse tailoring steps (e.g. hydroxylations, dehydrogenations, glycosylations, methylations, etc.) [7, 8]. Four genes are necessary for naringenin biosynthesis in a bacterium such as *Streptomyces albidoflavus* J1074: a tyrosine ammonia-lyase (TAL) converts L-tyrosine to *p*-Coumaric acid (*p*-CA), which is subsequently activated into coumaroyl-CoA by a 4-coumarate-CoA ligase (4CL). Then, one molecule of coumaroyl-CoA is condensed with three molecules of malonyl-CoA by chalcone synthase (CHS) to give rise to naringenin chalcone, which is then isomerized into naringenin via chalcone isomerase (CHI) [9]. Despite its outstanding properties, naringenin industrial production has been limited mainly to plant biomass extraction (mainly from citrus fruits), a process that requires high amounts of starting material, with a low productivity and a complex mixture of secondary metabolites generated, which makes the final purification a difficult task. In this regard, utilization of other biotechnological platforms, such as microbial-based expression systems, offers a promising alternative. *Escherichia coli, Saccharomyces cerevisiae, Streptomyces* spp. and *Yarrowia lipolytica* are some of the microorganisms that have been genetically engineered for the heterologous production of naringenin and other flavonoids [7, 10]. However, flavonoid production levels in these engineered microbes are low, which makes it necessary to further optimize the strains or their cultivation conditions in order to turn them into real microbial cell factories able to meet the industrial requirements. Among other microorganisms used for heterologous flavonoid production, *Streptomyces* spp. (well known as producers of multiple bioactive compounds) stand out as the most appropriate hosts, as they possess a complete repertoire of precursors, cofactors, and enzymes [11].

The naringenin biosynthesis pathway in a microbial host normally uses L-tyrosine and malonyl-CoA as initial precursors, in contrast to plants, where L-phenylalanine is usually the initial amino acid [10].

The cytoplasmic availability of precursors and co-factors constitutes the main bottleneck for naringenin heterologous production in bacteria. This issue can be addressed at fermentation and/or strain levels. Regarding fermentation conditions, a general approach consists of testing different production media, as culture medium composition optimization has been shown to affect at a great extent final naringenin heterologous production titers [12]. Such strategy to improve the production of flavonoids (or other natural compounds) has been proven to be highly successful in microorganisms with complex regulation, such as *Streptomyces* spp [13, 14]. Besides, medium composition can be modified by the addition of exogenous flavonoid precursors such as *p*-CA or L-tyrosine, which are bioavailable for the cell metabolism.

Also, in the case of malonyl-CoA, other feeding strategies have been adopted in order to enhance the intracellular pool of this precursor, such as the addition of the fatty acid synthase inhibitor cerulenin. This compound represses both FabB (3-oxoacyl-(acyl-carrier-protein) synthase (I)) and FabF (3-oxoacyl-(acyl-carrier-protein) synthase (II)) [15, 16], thus limiting the amount of malonyl-CoA that is used for the biosynthesis of fatty acids and therefore increasing the cytoplasmic pool of malonyl-CoA available for the biosynthesis of secondary metabolites, such as flavonoids in this case [17]. Another strategy is based on the additive feeding with the precursor of malonyl-CoA, malonate. However, this approach normally requires genetic modifications in order to incorporate a novel carbon assimilation pathway coded by the *matBC* operon, composed of the genes for malonate importer MatC and the malonyl-CoA synthase MatB [18]. The intracellular pool of malonyl-CoA can also be increased by modifying cellular pathways to channel carbon flux towards this precursor. As malonyl-CoA is an essential building block to produce fatty acids and phospholipids, its metabolism can be genetically engineered in order to favor its flux towards flavonoids biosynthesis [19]. However, these genetic modifications affecting primary metabolism building blocks must be cautiously applied in order to preserve the normal growth of the cell.

Also, some microorganisms, such as *Streptomyces* spp., possess endogenous biosynthetic gene clusters (BGCs) directing the biosynthesis of native secondary metabolites such as polyketides, which consume high amounts of cytosolic malonyl-CoA and other cofactors, therefore competing with the heterologous production of flavonoids, which needs the same precursors. Thus, removal of these native BGCs has been proven as a very successful strategy for diverting malonyl-CoA pools towards other targeted compounds, such as flavonoids, without altering neither the cell fitness nor the growth rate [9, 18, 19].

Apart from the modifications of host metabolic fluxes mentioned above, further improvements could be achieved by manipulating genes of naringenin BGC, such as testing alternative enzymes from different sources, or a proper balancing of enzymatic functions (adjusting the copy number of the different genes coding for naringenin BGC), as well as increasing the gene copy number. These approaches have shown positive results in different hosts, such as *E. coli* or *S. cerevisiae* [17, 20].

In this work, we have combined an array of metabolic engineering strategies in a stepwise manner (culture medium optimization, deletion of three chromosomal gene clusters, duplication of the naringenin BGC, heterologous expression of *matBC*, feeding with different flavonoid precursors or fatty acid biosynthesis inhibitors), in order to increase final naringenin heterologous production titers in the promising industrial host *S. albidoflavus* J1074.

## Results

### Effect of the culture medium on naringenin production

*S. albidoflavus* WT-NAR [9] is a *S. albidoflavus* J1074 wild type (WT) strain that harbors the naringenin BGC integrated into φC31 *attB* site. This strain was initially cultivated following our standard conditions for flavonoids production, which includes R5A as production medium [9, 21–23]. Cultivation was performed as described in Materials and Methods section, while the concentrations of naringenin and p-CA, as well as the biomass accumulation, were monitored every 24 h over 144 h.

**Fig. 1:**
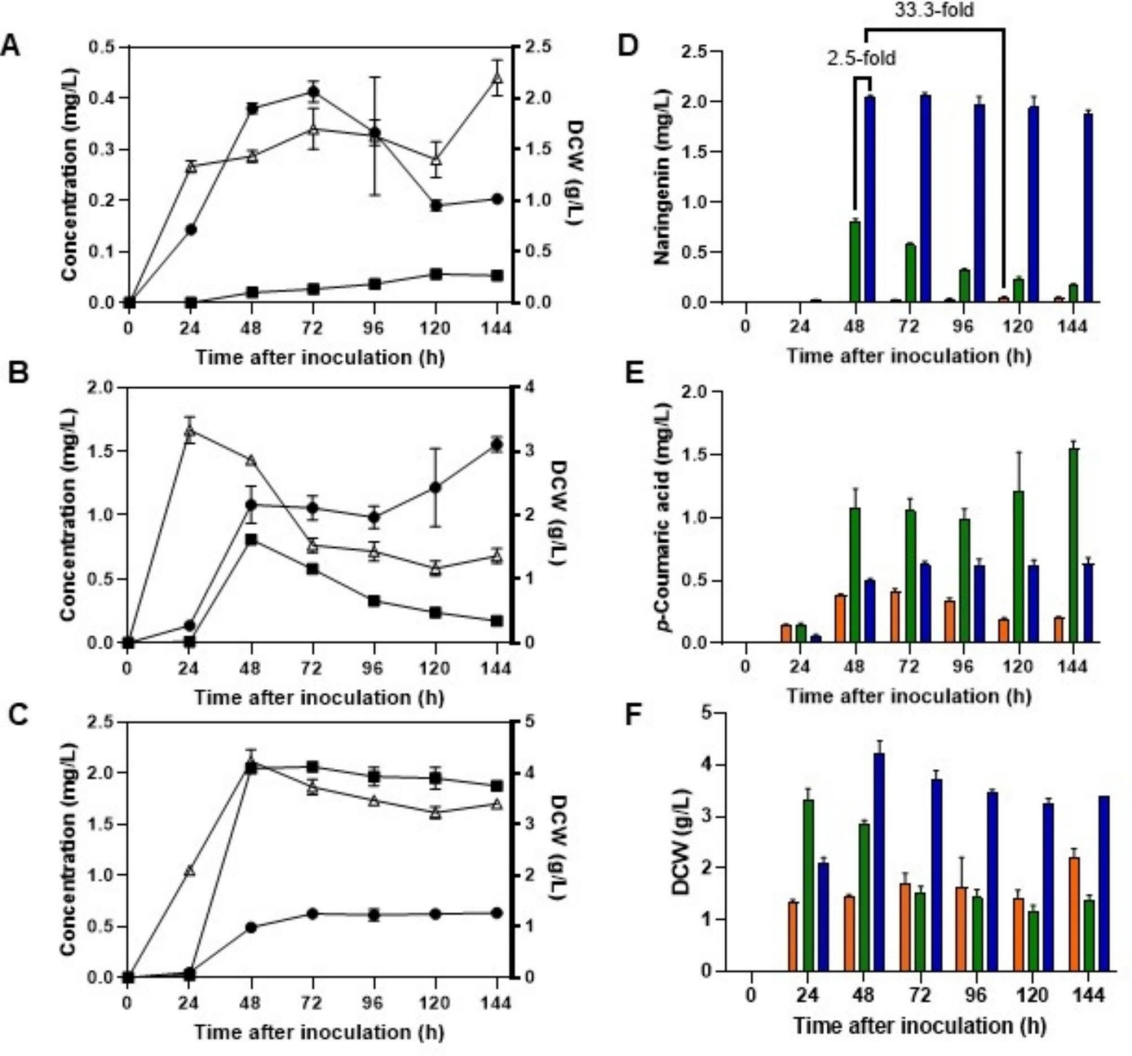
*** S. albidoflavus*** WT-NAR strain cultured in R5A, TSB and NL333. Time courses of naringenin production (■), *p*-Coumaric acid production (●), and growth (Δ) for ***S. albidoflavus*** WT-NAR in (A) R5A, (B) TSB, (C) NL333 culture media. Grouped time courses of (D) naringenin, (E) *p*-Coumaric acid, and (F) growth, in R5A (orange), TSB (green), and NL333 (blue). The folds of the increase in naringenin production are depicted. DCW: dry cell weight

According to our experience with heterologously produced flavonoids in this bacterium, naringenin titers are low, reaching 0.06 mg/L 120 h after the inoculation in R5A medium, concomitantly with a decrease in *p-*CA levels (Fig. 1A). Subsequently, levels of both molecules remain stable. The growth curve indicates an exponential phase until 24 h after inoculation, followed by stationary phase until 120 h after inoculation and a consequent increase in biomass that does not correlate with increased levels of naringenin nor *p*-CA in this culture medium (Fig. 1A).

As a first approach for improving naringenin titers, two other production culture media were tested: TSB (Fig. 1B), which has been used for native naringenin production in *S. clavuligerus* ATCC 27064 [13], and NL333 (Fig. 1C), which is a medium used for secondary metabolite production in *S. albidoflavus* J1074 [24].

*S. albidoflavus* WT-NAR showed improved performance in both of them compared to the R5A medium, achieving higher titers of naringenin, *p*-CA and biomass. Moreover, maximum production levels of naringenin in these media were achieved earlier than in R5A: after only 48 h of cultivation. This makes both media more suitable for an industrial naringenin production.

Regarding biomass accumulation, in TSB (Fig. 1B F), the exponential phase is, likewise the R5A, achieved 24 h after inoculation, but a decrease in this parameter is observed afterwards. In the case of NL333, the highest biomass was reached 48 h after inoculation (Fig. 1D F).

In the case of *p*-CA, in TSB medium, the maximum production concurs with naringenin highest peak, but in this case the *p*-CA levels are increased overtime, with a maximum *p*-CA concentration at the end of the cultivation (Fig. 1B and E). In the case of NL333 medium, *p*-CA showed a maximum at 72 h, with maintained levels towards the end of cultivation phase (Fig. 1C and E).

Finally, in the case of naringenin production, in R5A medium, its maximum takes place 120 h (Fig. 1A and D), whereas in TSB and NL333 media this maximum is achieved at 48 h (Fig. 1B C and 1D). These naringenin titers achieved 2 mg/L in NL333, entailing a 2.5-fold increase in the production compared to the maximum production in TSB (0.8 mg/L 48 h after inoculation) and a 33.3-fold increase compared to the maximum production in R5A (0.06 mg/L 120 h after inoculation) (Fig. 1D).

**Effect of the deletion of three native *S. albidoflavus *J1074 gene clusters on naringenin production**.

Our second approach to increase naringenin production titers was focused on removing *S. albidoflavus* J1074 native BGCs coding the biosynthesis of antimycins, candicidins and a cryptic PKS-NRPS compound. All three biosynthetic pathways require malonyl-CoA as a precursor. This strategy was successfully applied in our previous studies with *S. albidoflavus* J1074 producing heterologous eriodictyol [9].

**Fig. 2:**
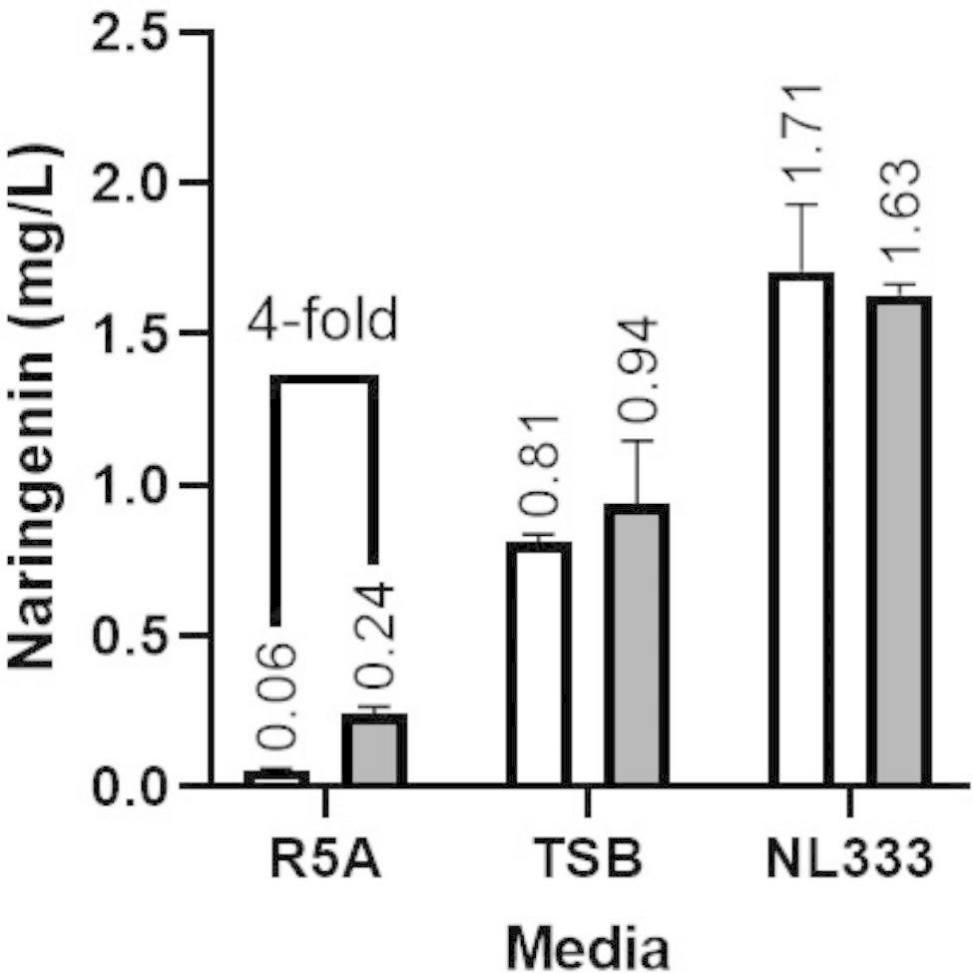
Effect of removing three endogenous BGCs encoding secondary metabolites that consume intracellular malonyl-CoA. *S. albidoflavus* WT-NAR (white) and *S. albidoflavus* UO-FLAV-002-NAR (grey) strains cultured in R5A, TSB and NL333 media. Naringenin concentration was measured 48 h after inoculation for TSB and NL333, and 120 h after inoculation for R5A. The folds of the increase in naringenin production are depicted

*S. albidoflavus* UO-FLAV-002-NAR [9] is a *S. albidoflavus* J1074 strain in which a chromosomal region containing the three abovementioned BGCs was replaced by the *matBC* genes from *Rhizobium trifolii* (under the control of the ermE* promoter) [25]. Moreover, *S. albidoflavus* UO-FLAV-002-NAR carries naringenin BGC integrated into фС31 *attB* site. Both, *S. albidoflavus* WT-NAR and *S. albidoflavus* UO-FLAV-002-NAR strains were cultivated in R5A, TSB and NL333 media, and naringenin production was measured at the maximum production time point for each media: 120 h for R5A and 48 h for TSB and NL333. No statistically significant differences were observed in TSB or NL333, but a 4-fold increase in naringenin production (0.24 mg/L vs. 0.06 mg/L) was observed when the new mutant strain *S. albidoflavus* UO-FLAV-002-NAR was cultivated in R5A culture medium compared to the initial strain *S. albidoflavus* WT-NAR strain (Fig. 2). These results validate the effectiveness of removing native BGCs encoding secondary metabolites which consume intracellular malonyl-CoA pool, as a strategy to increase final flavonoid production. Notably, NL333 culture medium remains optimal for the naringenin production in *S. albidoflavus* UO-FLAV-002-NAR.

### Effect of the additional copies of naringenin BGC on naringenin production

**Fig. 3:**
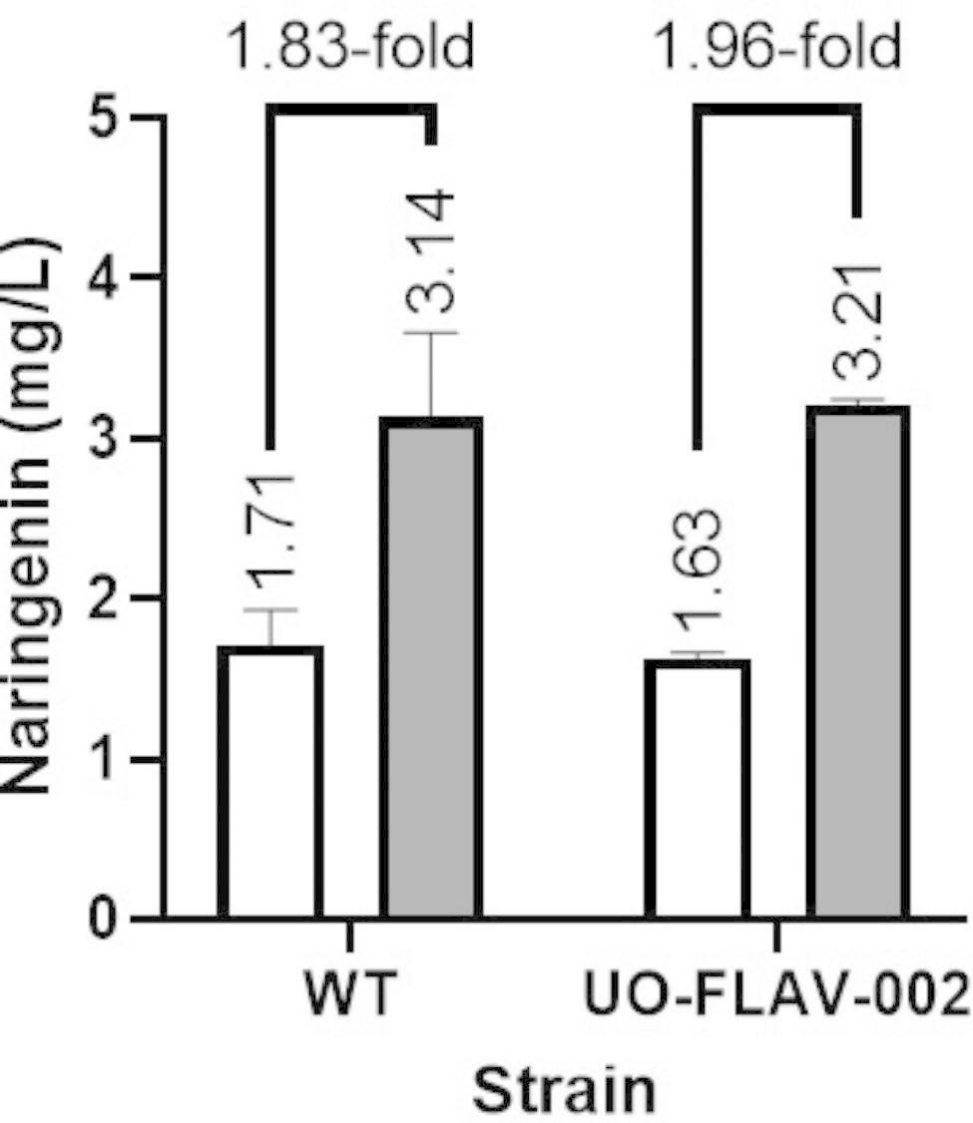
Effect of naringenin BGC duplication. *S. albidoflavus* WT and *S. albidoflavus* UO-FLAV-002 strains bearing one copy of the naringenin BGC (white) or two copies (grey), integrated into their chromosome. Naringenin concentration was measured 48 h after inoculation in NL333. The folds of increase in naringenin production are depicted

As a third approach, we introduced a second copy of naringenin BGC integrated in the bacterium chromosome. For this purpose, the naringenin BGC was assembled using Golden Standard and integrated into the φBT1 *attB* site of *S. albidoflavus* WT-NAR and *S. albidoflavus* UO-FLAV-002-NAR strains as described in Materials and Methods, to generate *S. albidoflavus* WT-NARNAR and *S. albidoflavus* UO-FLAV-002-NARNAR strains, respectively [26]. *S. albidoflavus* WT and *S. albidoflavus* UO-FLAV-002 strains bearing one or two copies of the naringenin BGC were cultivated in NL333 and naringenin production titers were measured 48 h after inoculation. In both cases, a similar increase in naringenin production was observed after the introduction of a second copy of the naringenin BGC: 1.83-fold (3.14 mg/L vs. 1.71 mg/L) in the *S. albidoflavus* WT strain, and 1.96-fold (3.21 mg/L vs. 1.63 mg/L) in the *S. albidoflavus* UO-FLAV-002 strain (Fig. 3).

### Effect of the heterologous expression of MatBC in combination with the additive feeding with malonate on naringenin production

**Fig. 4:**
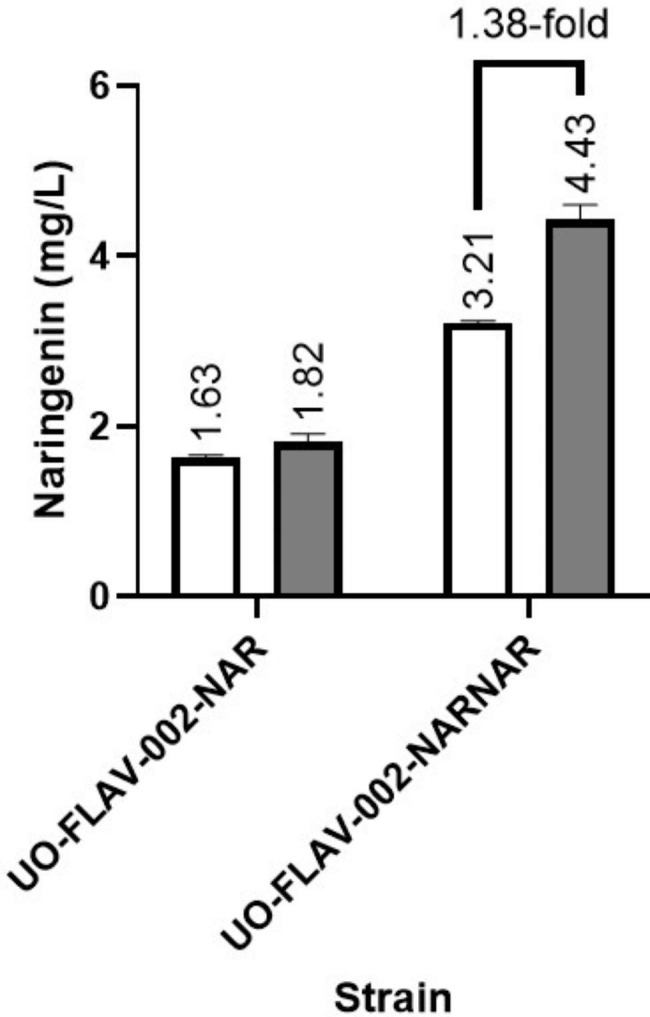
Effect of the heterologous expression of the MatBC enzymes upon malonate feeding. The *S. albidoflavus* UO-FLAV-002 strains bearing one or two copies of the naringenin BGC were cultured in NL333 (white) and NL333 with malonate 20 mM (grey). Naringenin concentration was measured 48 h after inoculation. The folds of the increase in naringenin production are depicted

The fourth approach we followed consisted of increasing intracellular malonyl-CoA pool by adding malonate to the culture medium. This precursor can be imported to the bacterial cell and converted to malonyl-CoA by the *matBC*-encoded enzymes. This operon was already integrated in *S. albidoflavus* UO-FLAV-002 chromosome, during the replacement of the native BGCs in this strain. The strains *S. albidoflavus* UO-FLAV-002-NAR and *S. albidoflavus* UO-FLAV-002-NARNAR were cultured in NL333 and in NL333 supplemented with 20 mM malonate. Naringenin production was measured in all cultures 48 h after incubation. No impact on naringenin production was observed after malonate feeding when one copy of naringenin BGC was present. However, when the strain containing two copies of the naringenin BGC was analyzed, a 1.38-fold increase (4.43 mg/L vs. 3.21 mg/L) was observed in the naringenin titers (Fig. 4), confirming the proper functioning of MatBC enzymes and validating the effectiveness of this strategy.

### Effect of feeding with the FAS inhibitor cerulenin on naringenin production

**Fig. 5:**
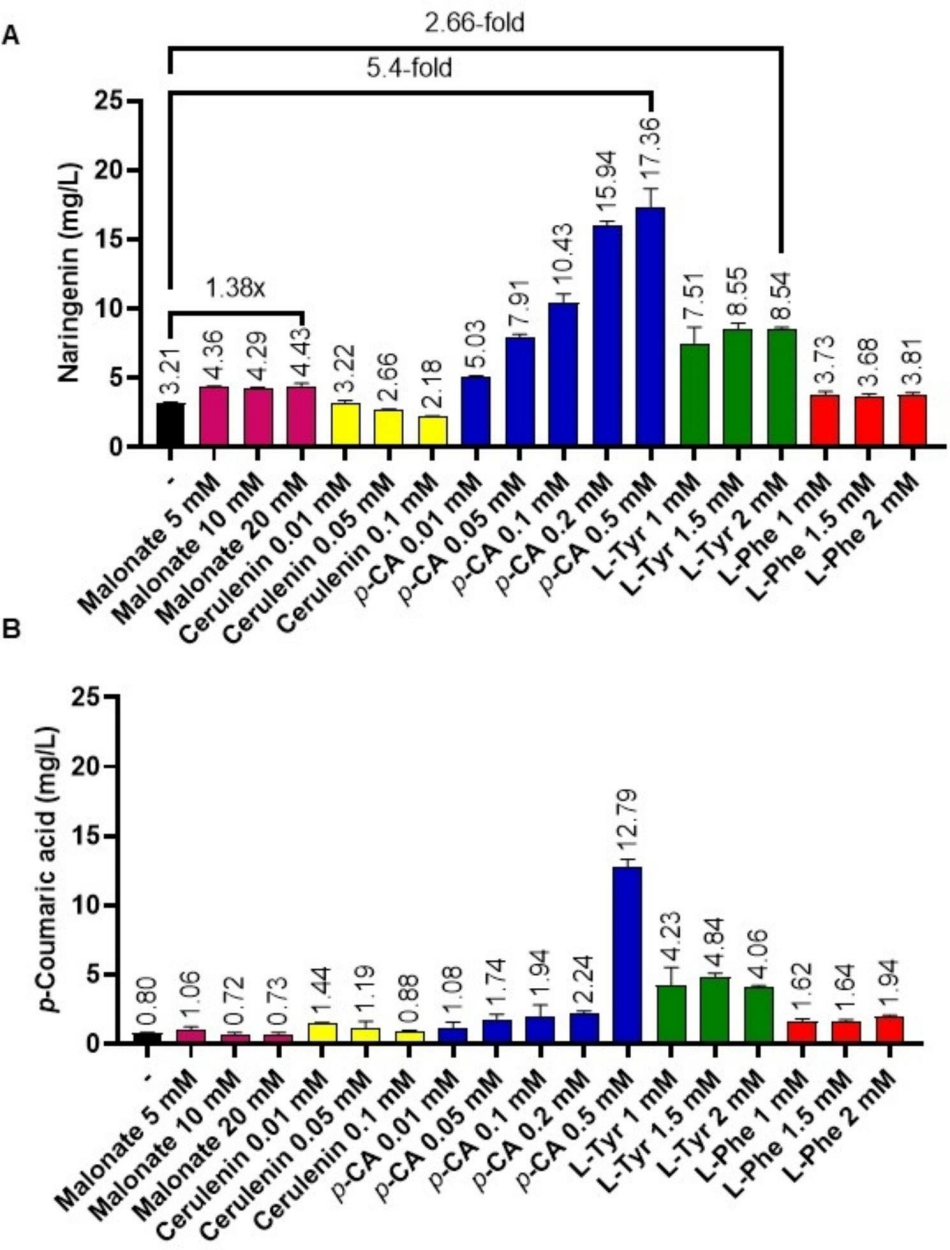
Effect of the feeding experiments. Cultures of *S. albidoflavus* UO-FLAV-002-NARNAR in NL333 culture medium were supplemented with different concentrations of malonate, cerulenin, *p*-Coumaric acid (*p*-CA), L-tyrosine (L-Tyr) and L-phenylalanine (L-Phe). Samples were taken 48 h after inoculation and assessed for (A) naringenin production and (B) *p*-Coumaric acid production. A control experiment in plain NL333 without feeding was included (-). The folds of the increase in naringenin production are depicted

To further increase naringenin production levels, the fatty acid synthase inhibitor cerulenin was added to the NL333 culture medium at different concentrations (0.01, 0.05 and 0.1 mM). However, no significant increase was observed in naringenin or *p*-CA titers at any of the tested concentrations (Fig. 5A and B). The antibiotic cerulenin is a non-competitive inhibitor of the fatty acid synthetases, which specifically acts by blocking the activity of β-ketoacyl thioester synthetase of this enzymatic complex, and therefore avoiding the use of malonyl-CoA [27].

### Effect of feeding with different precursors (malonate, L-tyrosine, L-phenylalanine, *p*-Coumaric acid) on naringenin production

In the last approach followed in the present study, the strain *S. albidoflavus* UO-FLAV-002-NARNAR was cultured in NL333 medium supplemented with different precursors at various concentrations: (i) malonate 5, 10 and 20 mM; (ii) *p*-CA 0.01, 0.05, 0.1, 0.2 and 0.5 mM; (iii) L-tyrosine 1, 1.5 and 2 mM; (iv) L-phenylalanine 1, 1.5 and 2 mM. A triplicate control experiment in plain NL333 culture medium was included. Samples were taken 48 h after inoculation and naringenin and *p*-CA concentrations were measured by HPLC-DAD.

As mentioned above, a 1.38-fold increase in naringenin concentration was observed when 20 mM malonate was added to the culture medium. This increase was similar to the increase achieved at lower concentrations of malonate (5 mM or 10 mM), suggesting that 4.43 mg/L is the maximum amount of naringenin that can be obtained after malonate feeding in the assayed conditions (Fig. 5A).

Regarding *p*-CA feedings, a dose-dependent response of naringenin production was observed, with a maximum naringenin titer of 17.36 mg/L accomplished using 0.5 mM of *p*-CA added in the culture medium. This implies a 5.4-fold increase in naringenin production compared to the control conditions without feeding (Fig. 5A). Regarding intracellular *p*-CA levels, this precursor is accumulated in higher amounts when 0.5 mM of *p*-CA is used in the feeding, achieving 12.79 mg/L of intracellular levels (Fig. 5B), suggesting that higher concentrations of *p*-CA supplemented to the culture medium would not lead to higher titers of naringenin.

When L-tyrosine was fed to the culture medium, a similar increase in naringenin concentration was observed at all the tested concentrations, achieving 8.54 mg/L at 2 mM of supplemented L-tyrosine, which constitutes a 2.66-fold increase compared to the control conditions (Fig. 5A). However, no increase was observed when the precursor added to the culture was L-phenylalanine (Fig. 5A). This precursor was selected to indirectly increment the L-tyrosine intracellular pools, as it has been shown to positively regulate L-tyrosine biosynthesis in *S. refuineus* [28] and its supplementation to the medium has led to higher titers of naringenin in *S. clavuligerus* ATCC 27064 [13].

Regarding the intracellular *p*-CA levels, it was observed that the concentration of this precursor remained below 2.24 mg/L at most of the assayed conditions, with the exception of the feeding with *p*-CA 0.5 mM and all L-tyrosine tested concentrations (1 mM, 1.5 mM and 2 mM) (Fig. 5B). Interestingly, and in contrast to *p*-CA feeding experiments (12.79 mg/L of intracellular *p*-CA after 0.5 mM *p*-CA feeding), intracellular *p*-CA concentrations when L-tyrosine was added to the medium were maintained at the same levels (between 4.06 and 4.23 mg/L) at all tested L-tyrosine concentrations, implying that *p*-CA accumulation in this case was probably due to different reasons than in the case of *p*-CA feeding.

**Fig. 6:**
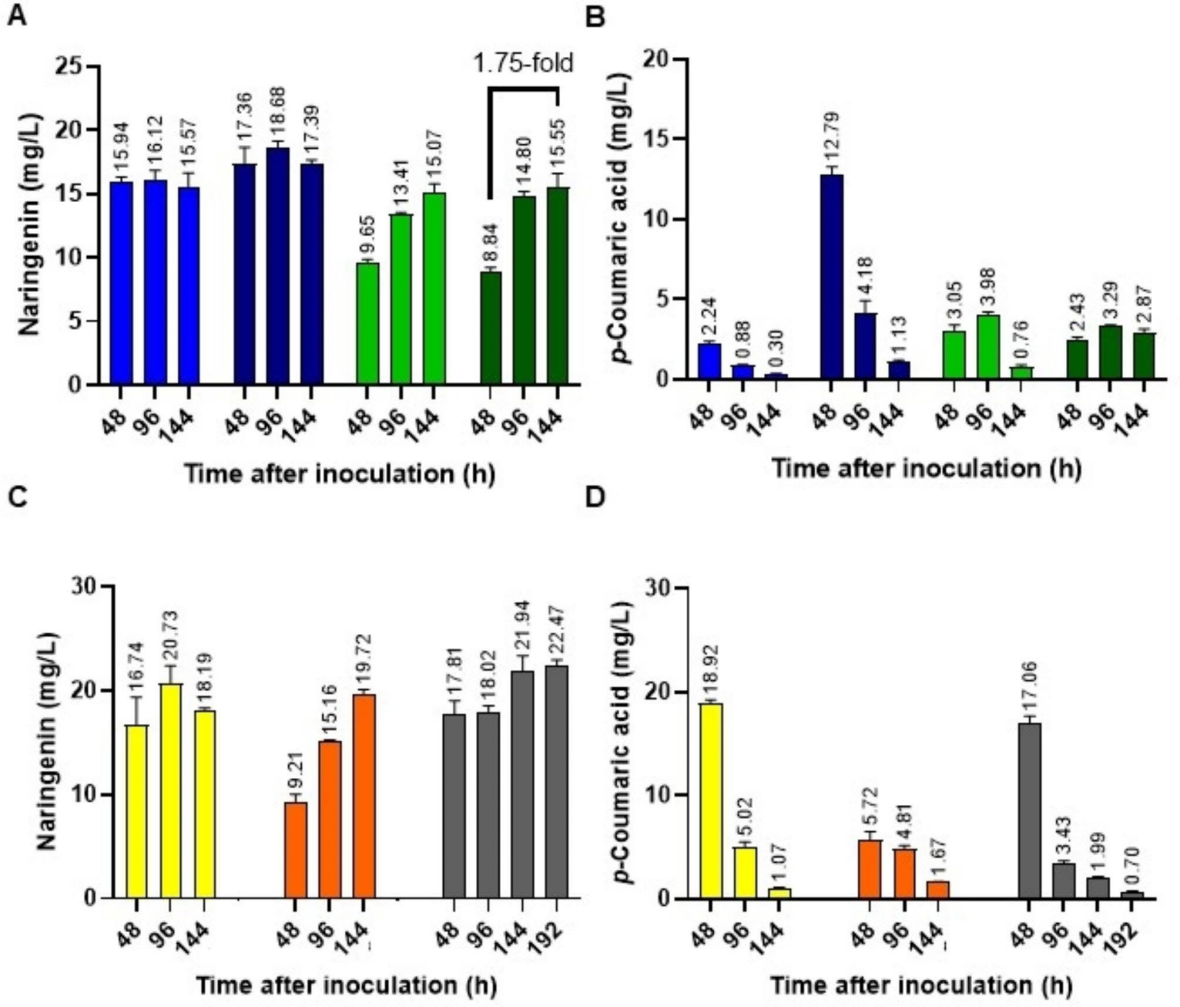
Effect of feeding experiments along incubation times. Time courses of feeding experiments of *S. albidoflavus* UO-FLAV-002-NARNAR with *p*-CA 0.2 mM (light blue), *p*-CA 0.5 mM (dark blue), L-Tyr 1.5 mM (light green), and L-Tyr 2 mM (dark green). (A) Naringenin concentration, (B) *p*-Coumaric acid concentration. Time courses of feeding experiments with *p*-CA 0.5 mM + malonate 20 mM (yellow), L-Tyr 2 mM + malonate 20 mM (orange), and *p*-CA 0.5 mM + L-Tyr 2 mM + malonate 20 mM (grey). (C) Naringenin concentration, (D) *p*-Coumaric acid concentration. The folds of the increase in naringenin production are depicted

Taking into account that L-tyrosine is a precursor converted to *p*-CA by the action of the TAL enzyme, we wondered if this accumulated *p*-CA might be later converted to naringenin and, therefore, higher levels of naringenin might be observed at later incubation times. Following this reasoning, new cultures were stablished adding *p*-CA 0.2 mM, *p*-CA 0.5 mM, L-tyrosine 1.5 mM or L-tyrosine 2 mM. Samples were taken every 48 h over 144 h total incubation, and naringenin and *p*-CA levels were measured by HPLC-DAD. In these experiments, higher titers of naringenin were observed at 96 and 144 h after feeding with L-tyrosine at both concentrations, with a maximum of 15.55 mg/L of naringenin recovered 144 h after inoculation in NL333 with L-tyrosine 2 mM (Fig. 6A). Thus, a 1.75-fold increase in naringenin titer was achieved after feeding with L-tyrosine 2 mM at 144 h incubation time in comparison with 48 h incubation. The overall increase achieved with L-tyrosine 2 mM feeding compared to the control without feeding was 4.8-fold. When *p*-CA was added to the culture medium, the maximum yield of naringenin was reached 96 h after incubation with p-CA 0,5 mM. This a 5.8-fold increase compared to the control without feeding (Fig. 6A). With respect to intracellular *p*-CA levels, a decrease in this precursor was observed overtime in the case of the *p*-CA feedings. In the case of L-tyrosine feedings, the *p*-CA intracellular levels were maintained until 96 h and decreased afterwards (in the case of 1.5 mM L-tyrosine feedings), or remained constant at every time point when 2 mM L-tyrosine was added to the culture (Fig. 6B).

Finally, another set of feedings were carried out combining different precursors: (i) *p*-CA 0.5 mM + malonate 20 mM; (ii) L-tyrosine 2 mM + malonate 20 mM; (iii) *p*-CA 0.5 mM + L-tyrosine 2 mM + malonate 20 mM. Naringenin and intracellular *p*-CA levels were measured every 48 h over 144 h (for the two first conditions), or over 192 h (for the last condition). In the case of *p*-CA and malonate joint feeding, naringenin and *p-*CA yields overtime showed the same trend as with *p*-CA alone (Fig. 6C and D). However, when malonate was combined with L-tyrosine, there was an increase in naringenin concentration 144 h after inoculation, reaching 19.72 mg/L (Fig. 6C). Accordingly, and in contrast to adding L-tyrosine 2 mM solely, a decrease in the *p*-CA levels was observed at the same time point (Fig. 6D). When *p*-CA, L-tyrosine and malonate were fed to the cultures, naringenin concentration overtime followed a combined trend between *p*-CA and L-tyrosine individual feedings. Thus, a high concentration of naringenin was observed at initial time points (48 and 96 h) and these titers increased afterwards (144 and 192 h), reaching 22.47 mg/L at 192 h after inoculation (Fig. 6C), which was the maximum concentration of naringenin achieved during this work.

## Discussion

The heterologous production of various flavonoids has been achieved by our research group in the industrially relevant microbial host *Streptomyces albidoflavus* J1074 [9, 21–23]. However, the general titers have remained rather low, with yields usually below 1 mg/L. In this work, we have focused our efforts in applying different metabolic engineering strategies in a combinatorial way, in order to enhance the naringenin yields in *S. albidoflavus* J1074.

The major improvement in the naringenin titers was achieved by culture media screening. Three media were tested: R5A, TSB and NL333, and among them, NL333 showed the best production titers, yielding 2 mg/L naringenin after 48 h of cultivation, which implies a 33.3-fold increase in naringenin production with respect to the 0.06 mg/L achieved in the standard culture conditions using R5A medium.

Malonyl-CoA was proven as a limiting factor for naringenin production in R5A, as removing three BGCs coding for biosynthetic pathways consuming malonyl-CoA led to a 4-fold improvement in naringenin yields. However, this behavior was not observed in TSB nor NL333 media. There are some possible explanations for this observed phenotype. On one hand, malonyl-CoA levels might not be a current bottleneck to naringenin production in TSB nor NL333. On the other hand, regardless of malonyl-CoA being a limiting factor, if neither of the three BGCs are expressed in these culture conditions, their deletion would not have any effect on the intracellular malonyl-CoA levels.

In the case of the NL333 culture medium, we have determined that the copy number of the naringenin BGC (integrated in the bacterial chromosome) was actually a bottleneck for naringenin production, rather than the lack of enough intracellular malonyl-CoA precursor, as nearly a 2-fold increase in naringenin levels was detected when a second copy of naringenin BGC was integrated into both *S. albidoflavus* WT-NAR or *S. albidoflavus* UO-FLAV-002-NAR chromosomes.

Moreover, when *S. albidoflavus* UO-FLAV-002-NAR cultures in NL333 were fed with malonate, no increase was observed in the final naringenin levels. However, when this malonate feeding experiment was carried out in *S. albidoflavus* UO-FLAV-002-NARNAR, a 1.38-fold increase in naringenin yields was observed, indicating that, after introducing a second copy of naringenin BGC, malonyl-CoA becomes a bottleneck for the production of this flavonoid.

Further experiments were carried out, adding cerulenin to NL333 culture medium, in order to increase malonyl-CoA intracellular pools by inhibiting the fatty acid synthase activity (an important consumer of malonyl-CoA in the cell), but this approach showed no increase in the final naringenin yields. In fact, a certain dose-dependent decrease in naringenin levels might be observed (Fig. 5A), despite not being statistically significant. Although other studies reported positive effects on naringenin production by adding cerulenin to other host fermentations, this opposite result obtained in *S. albidoflavus* J1074 is not surprising taking into account that cerulenin is able to bind to CHS [29], and that this compound has been proven to inhibit the biosynthesis of other malonyl-CoA-derived polyketides, such as candicidin in *S. griseus* [30]. Moreover, cerulenin has been shown to reduce *E. coli* biomass while increasing flavonoid yields [18], but this effect was not observed neither with *S. griseus* nor in our studies with *S. albidoflavus* J1074 (data not shown). Therefore, rather than exerting a great impact on the fatty acid biosynthesis, cerulenin might be binding to naringenin CHS and therefore blocking its biosynthesis in *S. albidoflavus* J1074.

Finally, the precursor L-tyrosine and its derivative *p*-CA were detected as the main bottlenecks for naringenin production by *S. albidoflavus* UO-FLAV-002-NARNAR cultured in NL333 medium (Fig. 5A). Feedings with these two precursors led to similar naringenin increase: a 5.8-fold at 96 h of cultivation when 0.5 mM *p*-CA was added to the medium, and a 4.8-fold at 144 h of cultivation when 2 mM L-tyrosine was added to the medium (Fig. 6A). Regarding this, *p*-CA feedings rendered higher naringenin yields in a shorter cultivation time. However, it is important to highlight the higher cost *p*-CA has with respect to L-tyrosine or L-phenylalanine. Feedings with various concentrations of L-phenylalanine were also conducted in order to enhance the L-tyrosine intracellular pools. However, no enhancement in naringenin yields was observed (Fig. 5A). As L-tyrosine indeed constitutes a bottleneck for final naringenin titers, we propose that for some reason, the exogenous addition of L-phenylalanine in our culture conditions are not leading to higher supply of L-tyrosine, maybe because externally added L-phenylalanine is deviated towards other catabolic pathways in *S. albidoflavus* J1074.

## Conclusions

In summary, the combination of different metabolic engineering strategies in *S. albidoflavus* J1074 in order to enhance the heterologous production of naringenin has led to an overall 374.5-fold increase in naringenin production, from the initial 0.06 mg/L obtained by cultivating the *S. albidoflavus* WT strain with one copy of the naringenin BGC integrated into its chromosome and using R5A (over 120 h incubation time), to a final 22.47 mg/L achieved by cultivating the mutant *S. albidoflavus* UO-FLAV-002 strain with two copies of naringenin BGC integrated into its chromosome, using NL333 supplemented with 20 mM malonate, 2 mM L-tyrosine and 0.5 mM *p*-CA (see Supplementary Figure S1 and S2).

Importantly, this work perfectly exemplifies the great influence that fermentation conditions have over the final yield of a targeted compound, even determining different precursors bottlenecks. Moreover, once one bottleneck is identified, the effectiveness of the strategy applied to address the same issue greatly varies and cannot be predicted beforehand. We believe that this work will serve to further develop *S. albidoflavus* J1074 as a flavonoid microbial cell factory.

## Materials and methods

### Bacterial strains, plasmids and culture conditions

All strains and plasmids used in this study are listed in Table 1. *E. coli* strains were grown in Tryptic Soy Broth (TSB, VWR, Barcelona, Spain) or on TSB agar plates. *S. albidoflavus* J1074 and derivatives were grown at 30 ºC in Yeast Extract-Malt Extract 17% (w/v) sucrose (YEME) for protoplasts preparation, and Bennett medium for sporulation [31]. Culture media were supplemented with the corresponding antibiotics (ampicillin 100 µg/ml, apramycin 50 µg/ml, thiostrepton 50 µg/ml on solid and 5 µg/ml in liquid medium) and reagents (X-Gal 40 µg/ml), when necessary.

For flavonoid production, *S. albidoflavus* J1074 spores were quantified and 10^6^ spores/mL were inoculated into R5A [32], TSB (VWR, Barcelona, Spain) or NL333 [24] culture media. NL333 pH was adjusted to 7.2. Cultures were incubated at 30 ºC and 250 revolutions per minute (rpm). Feeding experiments were carried out by adding the precursors to the medium at the same time that spores were inoculated.

**Table 1:** Plasmids and strains used in this work (Ap^R^: ampicillin-resistance; Tsr^R^: thioestrepton-resistance; Am^R^: apramycin-resistance)

Plasmid	Use in this work	Reference or source
pSEVAUO-M21703	φBT1, Ap^R^-Tsr^R^. Level 2 MoClo receptor vector for naringenin BGC cloning	(9)
pSEVAUO-M21102-TAL	φBT1, Ap^R^. Level 1 MoClo donor plasmid containing *P*_ermE*_-*TAL*	(9)
pSEVAUO-M21202-4CL	φBT1, Am^R^. Level 1 MoClo donor plasmid containing *SF14*-*4CL*	(9)
pSEVAUO-M21302-CHS	φBT1, Am^R^. Level 1 MoClo donor plasmid containing *SP25*-*CHS*	(9)
pSEVAUO-M21402-CHI	φBT1, Am^R^. Level 1 MoClo donor plasmid containing *SP43-CHI*	(9)
pSEVAUO-M21703-NarBGC	φBT1, Ap^R^-Tsr^R^. It harbors the naringenin BGC	This work
**Strain**	**Use in this work**	**Reference or source**
*Escherichia coli* Top10	Routine sub-cloning and DNA propagation	Invitrogen
*S. albidoflavus* WT-NAR	*S. albidoflavus* J1074 with naringenin BGC integrated into φC31 site	(9)
*S. albidoflavus* UO-FLAV-002-NAR	*S. albidoflavus* UO-FLAV-002 with naringenin BGC integrated into φC31 site	(9)
*S. albidoflavus* WT-NARNAR	*S. albidoflavus* J1074 with two copies of naringenin BGC, one integrated into φC31 site and another integrated into φBT1 site	This work
*S. albidoflavus* UO-FLAV-002-NARNAR	*S. albidoflavus* UO-FLAV-002 with two copies of naringenin BGC, one integrated into φC31 site and another integrated into φBT1 site	This work

### Reagents, biochemicals and enzymes

All solvents used for solid phase extraction and HPLC-DAD analyses were LC-MS grade from either Sigma-Aldrich (Madrid, Spain) or VWR Chemicals (Barcelona, Spain). Restriction enzymes and T4 DNA ligase were purchased from Thermo Scientific (Madrid, Spain). *p*-Coumaric acid, L-phenylalanine and sodium malonate were purchased from Sigma-Aldrich (Madrid, Spain), L-tyrosine from Acros Organics (Thermo Fisher, Madrid, Spain), and cerulenin from Cayman Chemicals (Ann Arbor, Michigan, USA).

### Construction of plasmid pSEVAUO-M21703-NarBGC

Recombinant DNA techniques were performed following standard protocols [33]. Plasmid pSEVAUO-M21703-NarBGC was assembled by a Golden Standard reaction using plasmids [9] and protocols [34]. For this, the Level 2 Golden Standard plasmid pSEVAUO-M21703 was used as receptor plasmid and mixed with Level 1 donor plasmids pSEVAUO-M21102-TAL, pSEVA-UOM21202-4CL, pSEVAUO-M21302-CHS and pSEVAUO-M21402-CHI in a Golden Standard reaction using *Bbs*I as restriction enzyme. Colonies harboring assembled constructs were selected by blue-white screening adding X-Gal to a medium containing ampicillin. Right assembly of the construct was first checked by restriction-digest and then confirmed by sequencing.

### Generation of the ***S. albidoflavus*** WT-NARNAR and ***S. albidoflavus*** UO-FLAV-002-NARNAR strains

The ϕ-BT1 integrative plasmid pSEVAUO-M21703-NarBGC was introduced into *S. albidoflavus* WT-NAR and UO-FLAV-002-NAR chromosomes by protoplasts transformation, following the corresponding protocol [31], giving rise to strains *S. albidoflavus* WT-NARNAR and UO-FLAV-002-NARNAR, respectively. Positive colonies were selected on the basis of their resistance to thiostrepton and checked by PCR.

### Naringenin and *p*-Coumaric acid extraction, HPLC-DAD analysis and quantification

1 mL culture samples from the different clones (cultivated in 25 mL culture medium in 250 mL Erlenmeyer flasks) were extracted with acetone (for the cellular pellet) and ethyl acetate (for the culture supernatant), as previously described [9].

The analysis of present flavonoids was performed using HPLC-DAD. Separation was conducted in a 1260 Infinity (Agilent Technologies, Madrid, Spain) HPLC system, equipped with an analytical column Pursuit XRs C18 (50 × 4.0 mm, 5 μm, Agilent Technologies, Madrid, Spain) with the column temperature operated at 30 ºC. Mobile phases consisting of 0.1% trifluoroacetic acid (TFA) in water (A) and acetonitrile (B), were employed at a flow rate of 1 mL/min. Mobile phases were eluted using the following linear gradient elution program: 10–40% B at 0–10 min, 40-50% B at 10–30 min, 50-100% B at 30–40 min, and 100%-10% B at 40–50 min. Detection and spectral characterization of peaks were carried out with a photodiode array detector and the analysis performed with the Data Analysis 4.3 software (Bruker). All chromatograms were extracted at 280 nm.

Naringenin and *p*-CA were quantified by comparing the peak area with that of a known amount of an authentic compound, using a calibration curve. The production titers are given in mg/L and the mean value was calculated from three biological replicates in each case.

### Electronic supplementary material

Below is the link to the electronic supplementary material.

**Additional file 1. Figure S1**: Maximum naringenin production achieved in the different assayed conditions. Every sample was taken 48 h after inoculation unless stated otherwise. The folds of the increase in naringenin production are depicted.

**Additional file 2**. Strategies carried out in order to enhance naringenin production titers in S. albidoflavus J1074, including feeding with different precursors.

## Data Availability

All data generated or analyzed during this study are included in this published article.
