# Supplementary figures and images for: Metabolic engineering strategies for naringenin production enhancement in Streptomyces albidoflavus J1074
Source: Microb Cell Fact. 2023 Aug 29;22:167. doi: 10.1186/s12934-023-02172-5 (PMC10466684; doi:10.1186/s12934-023-02172-5)

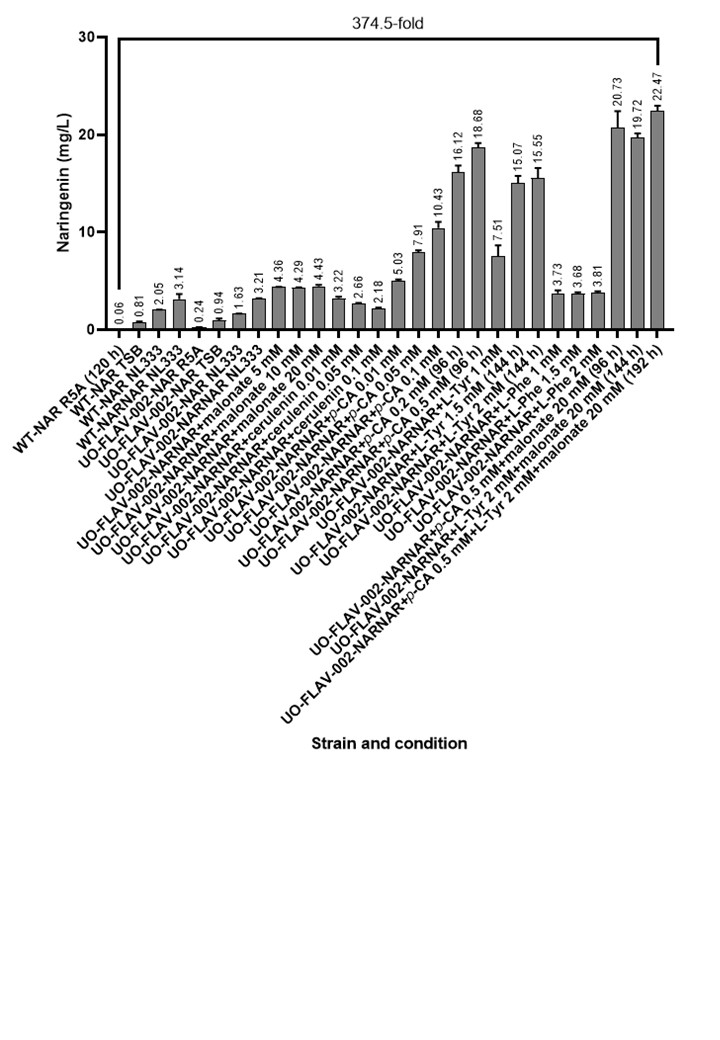

Supplement: Supplementary file 1 — Additional file 1. Figure S1: Maximum naringenin production achieved in the different assayed conditions. Every sample was taken 48 h after inoculation unless stated otherwise. The folds of the increase in naringenin production are depicted. [file 12934_2023_2172_MOESM1_ESM.jpg]

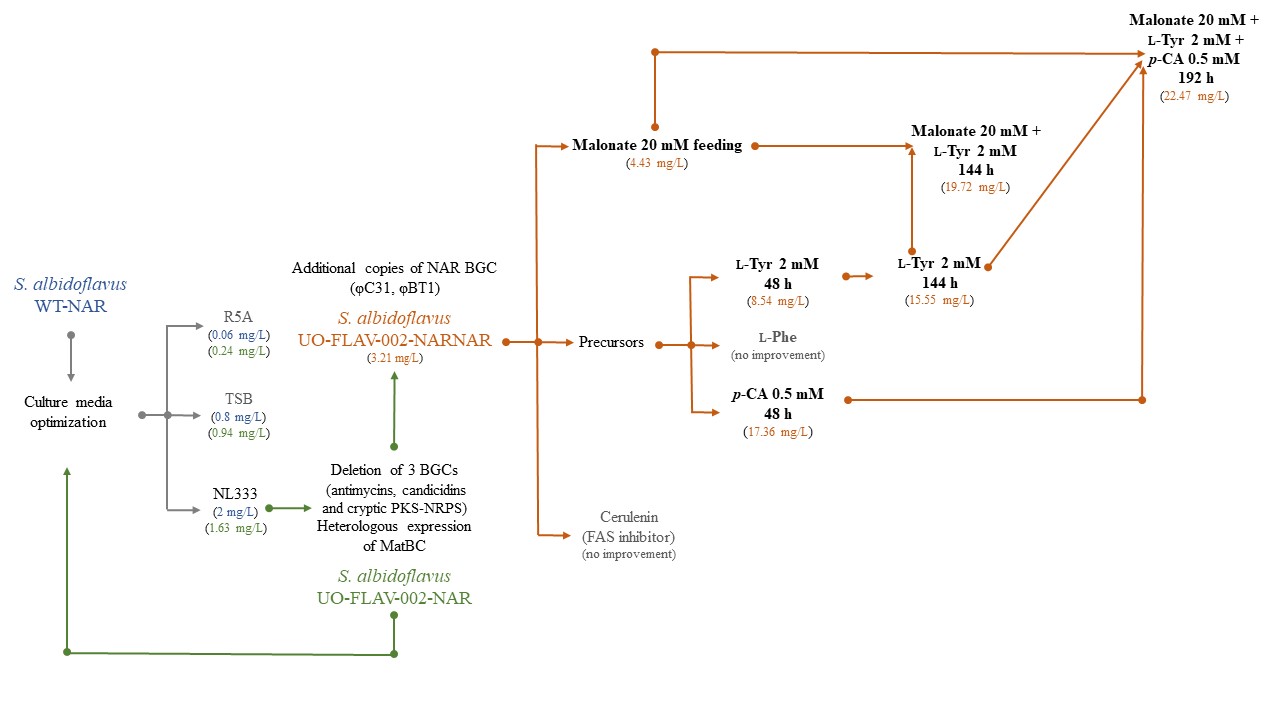

Supplement: Supplementary file 2 — Additional file 2. Strategies carried out in order to enhance naringenin production titers in S. albidoflavus J1074, including feeding with different precursors. [file 12934_2023_2172_MOESM2_ESM.jpg]
